# 

**Published:** 1892-12-10

**Authors:** 


CONTENTS.

Railway Servants' Vision . . . . . . . 161

Passing Topics.—Poor London! . . . . . . . . 162

Medical History from the Earliest Times. By E. T. Withington, M.A., M.B.Oxon. XXXV.—The Darkest Age (continued) . . . . . . . . . . . . 163

The Attitude of Great Writers Towards Disease. XVIII. Milton . . . . . . . . . . . . 164

Diet in Disease. By Mrs. Ernest Hart. IX.—Digestion (continued) . . . . . . . . . . 165

Everybody's Page . . . . . . . . . . 166

Annotations.—Hygiene and Caligraphy—Mirth in a Model Lodging House—See-Saw . . . . . . . . . . . 167

Notes and News . . . . . . . . . . . 168

Scraps and Gleanings . . . . . . . . . . 176

The Student of Medicine—Appointments, Vacancies,

The Hospital Clinic—

Liverpool Royal Infirmary.—The Treatment of Typhoid Fever . . . . . . . . . . . . 169

Royal Free Hospital.—The Treatment of Pleurisy . . . 170

Northampton General Infirmary.—The Treatment of Acute Rheumatism and Its Complications . . . . . . 171

The Practitioners' Bookshelf.—" A History of Medical Education " . . . . . . . . . . . 171

Books Received . . . . . . . . . . . . 172

The Institutional Workshop—

Within the Hospitals.—Edinburgh Royal Infirmary . . 173

Hospital Construction.—Victoria Hospital, Folkestone . 174

New Hospitals.—Royal Eye Hospital, Southwark . . 175

Hospital Opinion.—Paying Patients at Special Hospitals . 176

New Drugs, Appliances, and Things Medical . . 176

EXTRA SUPPLEMENT.—THE NURSING MIRROR.

En Passant.—Funeral Honours—Our Christmas Parcels—A Nurse for Seaham Harbour—Women's Convalescent Home Association—Glasgow Sick Poor and Private Nursing Association—Short Items—Belfast Nurses' Home and Training School—The Privy Council and Nurse Registration . . . . . lxxxiii

Lectures for Asylum Attendants. By William Harding, M.B. (concluded). X.—Entertainments . . . . lxxxiv

Merit and Modesty.—A Nurse's View . . . . . lxxxv

Notes on Novelties.—Nurses' Uniforms . . . . . lxxxv

A New Departure . . . . . . . . . . . lxxxv

Everybody's Opinion.—The Daily Walk—St. John's Maternity Home—The Nurses' Bookshelf . . . . . . lxxxvi

Examination Questions.—On Certain Points in the Nursing of Typhoid . . . . . . . . . . lxxxvii

Death in Our Ranks—Notes and Queries . . . . lxxxvii

For Reading to the Sick.—Bitterness . . . . . lxxxvii

Some Australian Experiences.—The Children's Hospital Brisbane . . . . . . . . . . . lxxxvii

